# Prediction of Drug Efficacy in Colon Cancer Preclinical Models Using a Novel Ranking Method of Gene Expression

**DOI:** 10.3390/cancers12010149

**Published:** 2020-01-08

**Authors:** Justine Fritz, Olivier Lefebvre, Aurore Fernandez, Jordane Schmidt, Dominique Bagnard

**Affiliations:** INSERM U1109, Microenvironmental Niche in Tumorigenesis and Targeted Therapy (MN3T Lab), University of Strasbourg, Labex Medalis, Fédération de Médecine Translationnelle, 67000 Strasbourg, France; jfritz@adaptherapy.com (J.F.); lefebvre@unistra.fr (O.L.); Aurore.Fernandez@chuv.ch (A.F.); jordane.schmidt@unistra.fr (J.S.)

**Keywords:** gene expression, drug efficacy, targeted therapy selection, molecular profiling

## Abstract

The presence of stromal cells in tumors is altering the significance of molecular profiling when using standard methods of gene expression quantification. We developed a novel normalization method to rank target gene expression in tumor samples by comparisons with reference samples representing the different cell types found in a tumor. The score for each target gene obtained after normalization, is aimed to be predictive of targeted therapies efficiency. We performed this qPCR analysis on human colorectal cancers to demonstrate the importance of reference samples to obtain accurate data and on a collection of patient-derived xenografted (PDX) colon tumors treated with Cetuximab (anti-EGFR) to demonstrate that the calculated EGFR score is predictive of Cetuximab efficacy. Interestingly, the score allowed to select an efficient treatment in a PDX model refractory to standard of care. This method is opening a novel way to predict targeted therapy efficiency which could be extended to several tumor types, and to unlimited target genes.

## 1. Introduction

A cancer is a dynamic disease due to an acquisition of genomic alterations triggering perturbations of signaling pathways that control, among others, cell growth, proliferation, apoptosis, or migration [1,2]. Due to the increasing research in this field, the mechanisms that govern these alteration processes are becoming increasingly understood. In fact, the generalized molecular characterization of tumors using multiple omics approaches has generated over the last 15 years a gold mine of data revolutionizing the current clinical practice [3]. Thanks to this new knowledge, a tumor is nowadays not only classified as usually by organ or cell type, but also according to molecular abnormalities and specific molecular characteristics [4,5,6]. While helping to understand the mechanisms of cancer onset and progression, the vast availability of information is strengthening the concept of personalized medicine for better and more precise diagnosis and prognosis or predicting treatment efficacy [7]. Hence, this concept is also concomitant with the development of targeted therapies differing from conventional chemotherapies, since they specifically target key players of the processes involved in neoplastic transformation of a tumor cell or in tumor progression [8]. The current challenge is to find the best adapted targeted therapy for a patient according to their specific tumor molecular characteristics. Patients can therefore eventually be classified into subgroups of responders or non-responders according to precise characteristics reflecting genetic alterations of molecular targets known to be responsible for the carcinogenesis process or leading to resistance to targeted therapies. The *KRAS* mutation is, for example, predictive of Cetuximab efficacy in colorectal cancer [9,10]. However, besides genomic instability in cancer cells, one major hurdle slowing the systematic use of such approaches in the daily clinical practice is intra-tumor heterogeneity. Indeed, it is well known that a tumor comprises several clonal expansions whose genetic inhomogeneity has been clearly revealed [11]. This intra-tumor heterogeneity is in part responsible to resistance to targeted therapies [12]. Moreover, cancer cells in solid tumors are surrounded by a complex cellular ecosystem made of endothelial cells, eventually normal cells, and several subtypes of infiltrating immune cells. Finally, profound modifications of the extracellular microenvironment are also contributing to tumor heterogeneity [13]. This complexity has been exemplified by Werb et al. comparing tumors as organs in organs [14]. Thus, when analyzing a tumor biopsy, it is mandatory to take into account the cellular complexity reflecting the potential enrichment of one particular cell type or may contain more non-tumor cells or may correspond to hypoxic/necrotic region associated with disorganized extracellular matrix. This conjunction of factors is highlighting the need to define a correct reference sample serving to calibrate the normal level of expression of a given gene in order to properly identify up or down-regulation in the pathological context. Generally, the expression data are produced using one or more housekeeping genes and eventually by comparison with the normal tissue when applicable such as in the WINTHER initiative [15]. Here, we decided to take into account the tumor complexity by comparing the level of the expression of a given gene determined in a tumor sample with its expression in the whole organ hosting the tumor (here the colon) and to take into account of the diversity of cell types in a tumor with its expression in the normal major cell populations constituting the organs (here colon epithelial cells, colon smooth muscle cells). To integrate the early modifications of the cellular composition in precancerous/low grade tumors we also compared expression levels with precancerous (here polyps) tissue samples. Each comparison was used to normalized the expression of target genes and the sum of these relative expression levels was then calculated in order to determine a global target gene expression level reflecting the real deregulation of the gene expression with regards to the different cellular components of the tumor. The expression scores were calculated here for a limited list of target genes arbitrary selected for their implication in tumor-associated processes and described as relevant targets in colorectal cancer (see Table 1). This includes genes involved in proliferation (*ITGB1*, *ERBB2*, *EGFR*, *MMP2*, *MMP9*, *PDGFR*), vascularization (*VEGFA*, *Flt1*, *KDR*), extracellular matrix remodeling (*MMP2*, *MM*P9), and all being the target of targeted therapies in clinical development or already in-use.

We conducted this proof of concept experiment on colorectal cancer (CRC) because, despite progress in diagnosis and treatment, CRC is still a deadly cancer with a 5-year net survival not exceeding 60% ([24]. In addition to microsatellite instabilities [25], mutations are sought to identify tumor subtypes. Overall, frequent alterations in colorectal cancers are a loss of *APC* (70%), mutations of *TP53* (30–50%), *KRAS* (35–40%), *BRAF* (5–10%), *NRAS* (3 to 5%), *PI3C*A (25%). In addition, *IGF2* is amplified in 30% of cases and *ERBB2* in 4% of cases [26]. Hence, the progressive evolution of CRC together with the accumulation of mutations and the existence of well-established targeted therapies represented an ideal model to challenge whether our normalization method independent of the mutational status of the tumor would modify our understanding of gene expression in such a complex and evolving cellular system. Using RNA samples from patient biopsies, we first showed the importance of the reference samples used to normalize data and to get relevant levels of expression of a given target gene. We then used 15 patient-derived xenograft tumor samples to prove the possibility to obtain a correlation of an appropriately normalized expression level of EGFR with the response to Cetuximab. The detailed analysis of this cohort also revealed that the method we developed allowed the stratification of responding and non-responding tumors. Hence, we used this method to evaluate in an animal model of CRC whether the administration of targeted therapies selected from the proposed ranking method could be used to select efficient targeted therapies in tumors derived from a non-responder patient. Strikingly, we were able to show in a preclinical model the predictive value of the proposed normalization process and calculated expression scores.

## 2. Results

### 2.1. Selection of a Normalization Process Taking into Account Tumor Complexity

**Figure 1:**
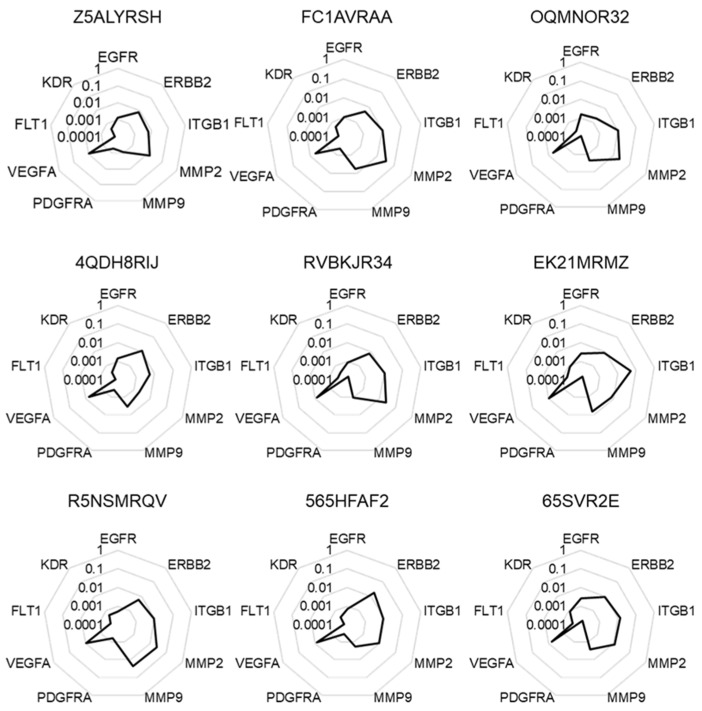
Molecular signatures without normalization. The different radar graphs are showing the molecular signature of each tumor sample when expression data were expressed according to the mean expression of two independent housekeeping genes *GAPDH* and *18S* (2^−ΔCt^).

We accessed to a set of RNA samples of nine human colorectal cancer biopsies from Bioserve tumor samples collection. After controlling RNA integrity, we performed RT-qPCR analysis to determine the expression levels of nine target genes (*EGFR*, *ERBB2*, *ITGB1*, *MMP2*, *MMP9*, *PDGFRA*, *VEGFA*, *FLT1*, and *KDR*). As seen in Figure 1, we obtained similar expression profiles for all biopsies when the data were expressed as 2^−ΔCt^ corresponding to the mRNA levels of target genes when compared to the mean expression of two different housekeeping genes (*GADPH* and *18S*) in the samples.

**Figure 2:**
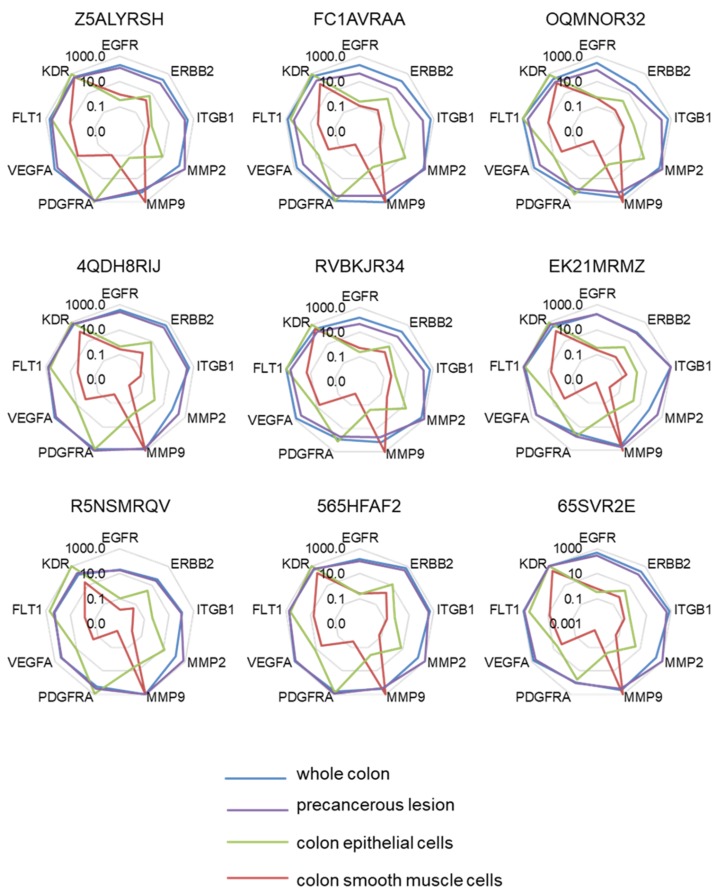
Molecular signatures after single normalization. The different radar graphs are showing the molecular signature of each tumor sample when expression data were expressed according to comparisons (2^−ΔΔCt^) with whole normal colon (blue line), precancerous lesion (purple line), colon epithelial cells (green line), or colon smooth muscle cells (red line).

Moreover, this approach was not able to identify significant expression level variations among the different genes. The mRNA levels were then compared to the one measured in a reference sample using the 2^−ΔΔCt^ formula (Figure 2). Here, the expression level of target genes was to this end searched in the whole normal colon, a precancerous colon lesion (Polyp), in human colonic smooth muscle cells, and in human colonic epithelial cells. Interestingly, the expression profiles of target genes appeared very different when taking into account these reference samples. The closest profile was obtained in comparison with the polyp reference sample, while marked differences were obtained in comparisons with smooth muscle cells.

A statistical analysis conducted in the nine independent samples confirmed significant variations of individual profiles when comparing the data obtained after the different types of normalization (Table 2).

**Figure 3:**
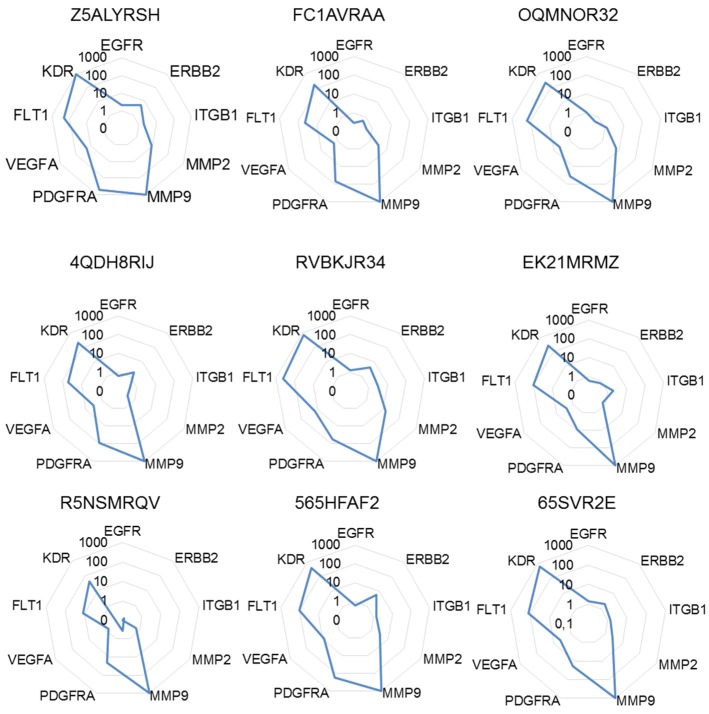
Molecular signatures after multiple normalizations. The different radar graphs are showing the molecular signature of each tumor sample when expression data were expressed as the sum of 2^−ΔΔCt^ further normalized to 1000, an arbitrary unit attributed to the gene with the highest sum of 2^−ΔΔCt^.

Hence, to take into account as much as possible this variability, we calculated for each target gene the sum of the 2^−ΔΔCt^ to obtain a full picture of the relative expression of target genes. The highest sum was then arbitrary set to 1000 in order to calculate a normalized score of expression ranking all target genes from this particular level of expression being the gene for which the deregulation (in this case upregulation) was seen with the highest amplitude. As depicted in Figure 3, the signature of target genes using the scores of expression was differing from the expression data determined prior to the normalization process or single normalizations. This calculation mode also demonstrated genes strongly upregulated in all patients (e.g., *MMP9*, *KDR*), while individual variations could be monitored for other genes (e.g., *MMP2*, *PDGFRA*, *VEGFA*).

**Figure 4:**
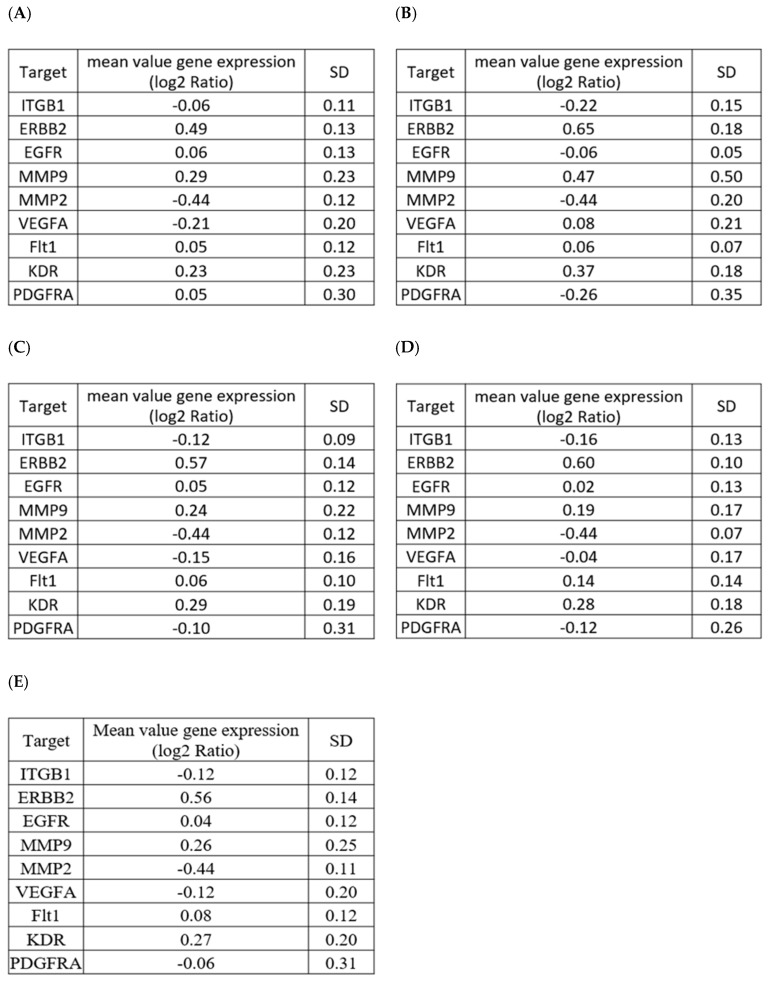
Data mining of target genes expression in human normal colon epithelial biopsies. Analysis of mean values of gene expression (log2 ratio) of selected target genes in (**A**) normal uninflamed sigmoid colon (*n* = 24), (**B**) normal uninflamed terminal ileum (*n* = 6), (**C**) normal uninflamed descending colon (*n* = 22), (**D**) normal uninflamed ascending colon (*n* = 17), and (**E**) all colon samples (*n* = 69). Data were extracted at the GEO platform (GEO access number GDS3268 from [27]).

To address whether one single sample is sufficient for each reference sample types, we performed data mining of target genes expression in human normal colon epithelial biopsies. As seen in Figure 4, we found that all target genes were expressed in a comparable amount in 69 different samples analyzed in a whole or as a function of their anatomical region (sigmoid colon, terminal ileum, descending or ascending colon). This suggested that at least for this proof of concept study, the use of one single reference sample is representative of a larger population.

**Figure 5:**
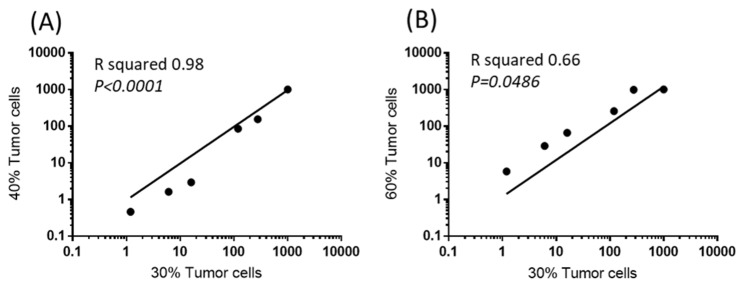
Sores of expression of 6/9 target genes as a function of tumor cell content. Cross correlation of the expression levels (scores) in a sample region containing 30% tumor cells with other regions of the same sample containing 40% (**A**) or 60% tumor cells (**B**).

Another important issue is the actual percentage of tumor cells in the tumor biopsies. Indeed, depending on the sampling, there might exist strong differences in the tumor cell contents. We were able to determine the scores of expression of 6/9 of the target genes (restricted to six genes due to limitation of sample size) from three different formaldehyde fixed paraffin embedded sections of a same tumor sample containing different percentage of tumoral cells (30%, 40%, 60%) certified by a pathologist (Service de Pathologie, Hôpitaux Universitaires Paris-Centre, Site Cochin, Paris, France). A cross correlation analysis revealed that the score of expression calculated for the different genes from the tumor sample with 30% tumor cells were similar to the ones determined from samples containing 40% or 60% tumor cells. A Spearman’s rank correlation coefficient (Rs) of 1 (*p* = 0.0028) reflected the strong correlation of the measurements. This demonstrated that the normalization method is highly sensitive and is applicable to tissue samples containing at least 30% of tumor cells (Figure 5).

### 2.2. The Normalized Score of Expression of EGFR Correlates with Cetuximab Efficacy

**Figure 6:**
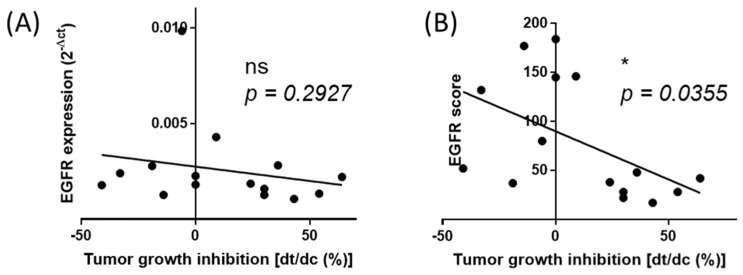
The normalized score of expression of EGFR correlates with Cetuximab (anti-EGFR) efficacy. (**A**) Correlation of the non-normalized expression of EGFR expression (2^−ΔCt^) with Cetuximab tumor growth inhibition in the cohort of 15 patient-derived tumor xenografts models. (**B**) Correlation of the EGFR score (sum of 2^−ΔΔCt^ further normalized to 1000) with Cetuximab tumor growth inhibition in the cohort of 15 patient-derived tumor xenografts models. dt%/dc% index is used as an exact value of Cetuximab efficacy. Spearman r analysis was applied.

We next used a cohort of 15 PDX colon cancer issued from the well-characterized collection published by Julien et al. [28]. Samples comprised tumors with various mutations and microsatellite instability (MSI) profiles to create a situation where any information extracted from our normalization process should not be dependent on these criteria (for details see Supplementary Table S1). RNA samples were collected and analyzed as described for the patient biopsies to calculate the score of expression, i.e., when data were expressed using the normalization process including the different reference samples. The PDX models used in this study have been previously extensively characterized and their response to Cetuximab is well documented. We used this information to determine the correlation between the expression levels of EGFR with Cetuximab efficacy (expressed by the percentage of tumor growth inhibition (dt/dc)%). As seen in Figure 6, there is no correlation between the raw expression levels (before normalization) of EGFR expression (2^−ΔCt^), thereby demonstrating that EGFR expression cannot be used to predict treatment efficacy if not appropriately determined (*p* = 0.2927). However, we found a significant correlation between the EGFR expression scores (normalized expression being the sum of 2^−ΔΔCt^) and Cetuximab efficacy (*p* = 0.0383). The highest scores corresponded to the strongest efficacy of Cetuximab. This result suggested that the EGFR expression level can predict drug efficacy only when determined using an appropriate method taking into account the complexity of tumors samples.

### 2.3. The Score of Expression of EGFR Can Be Used to Discriminate Responders Versus Non Responders

**Figure 7:**
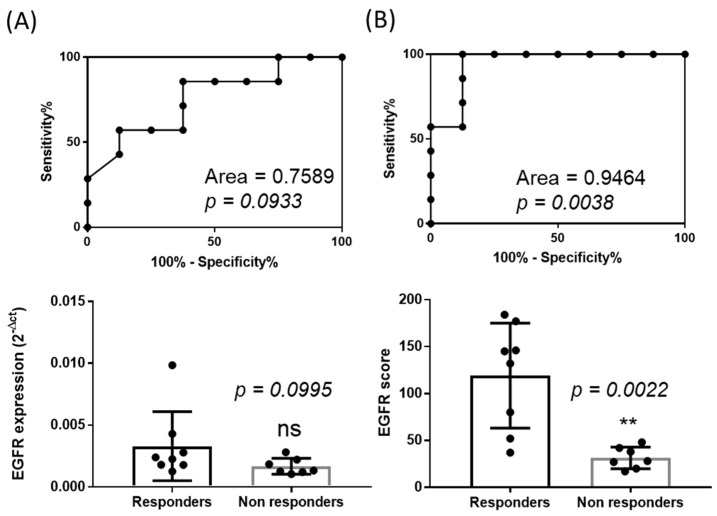
Discrimination of responders versus non-responders. (**A**) ROC curve analysis of the non-normalized expression of EGFR (2^−ΔCt^) according to the objective response to Cetuximab (defined as responder if (dt/dc)% < 10% and non-responder if >10%) in the cohort of 15 patient-derived tumor xenografts models. (**B**) ROC curve analysis of the normalized expression of EGFR (sum of 2^−ΔΔCt^ further normalized to 1000) according to the objective response to Cetuximab (defined as responder if (dt/dc)% < 10% and non-responder if >10%) in the cohort of 15 patient-derived tumor xenografts models. Median expression of EGFR before normalization (C, 2^−ΔCt^) or after normalization (D, sum of 2^−ΔΔCt^ further normalized to 1000). Mann-Whitney test (ns: not significant; ** *p* < 0.01).

Thanks to the systematic measurement of tumor growth upon Cetuximab treatments, we were able to classify the PDX models into eight responders (R, (dt/dc)% < 10%) and seven non responders (NR, (dt/dc)% > 10%) [23]. This allowed us to evaluate the performance of our normalization method in discriminating Cetuximab responding and non-responding tumors. As seen in Figure 7, a Receiver Operating Characteristic (ROC) curve analysis (Zweig and Campbell, 1993) showed an area under the curve of 0.95 (±0.06) demonstrating a strong and significant discrimination capacity (*p* = 0.0038) with a 95% confidence interval (0.83–1.06). Interestingly, an ROC curve analysis of non-normalized data (raw expression data) confirmed that EGFR level of expression if not appropriately determined is not predictive of the response to the drug (area of 0.76 ± 0.13, *p* = 0.1) with a 95% confidence interval (0.5–1.01). Consistently, the median of EGFR expression before normalization was similar for the two groups (0.00234 for R versus 0.00134 for NR, *p* = 0.1 Mann Whitney test), while the calculation of the scores of expression revealed a significantly higher median score for tumors of the responding tumor group (138.5 for R versus 28 for NR, *p* = 0.002 Mann Whitney test).

### 2.4. The Highest Score of the Normalized Signature is Predictive of Drug Efficacy

**Figure 8:**
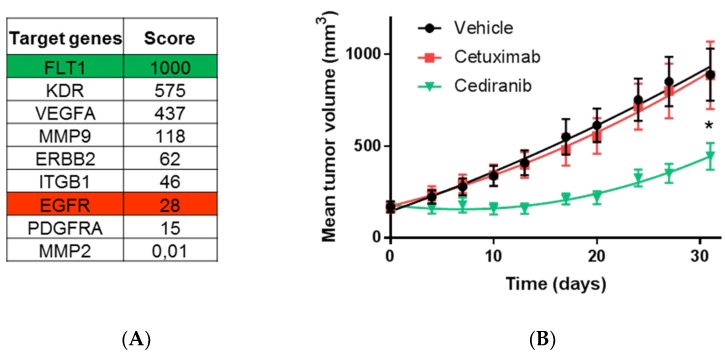
The highest score of the normalized signature is predictive of drug efficacy. (**A**) Ranking of target genes obtained after normalization of the expression data in PDX model CR IC 028M-P3. (**B**) Evaluation of the predictive value of the scores in the PDX model CR IC 028M-P3 known to be refractory to anti-EGFR treatment (Cetuximab). Mean tumor volumes measured during the time of treatment with Cetuximab (red line) predicted to be inefficient, Cediranib (green line) predicted to be the most efficient drug among the selected target of the signature or vehicle (black line) used as a positive control of tumor growth. Student *t* test analysis (ns: not significant; * *p* < 0.05).

To address whether this scoring method could help identifying efficient drugs on refractory tumors, we selected one of the PDX model previously shown to be resistant to Cetuximab. Tissue blocks were grafted in mice and the molecular signature of the nine target genes was established from a sentinel tumor sample collected two days before the administration of treatments. As seen in Figure 8, the scoring procedure revealed that FLT1 obtained the highest score (1000 points) while KDR and VEGFA were also exhibiting high scores (575 and 437, respectively). The EGFR score was 28, being at the bottom of the list. We decided to test whether Cediranib, a potent inhibitor of FLT1/KDR could trigger anti-tumor effect in this Cetuximab-resistant model. Hence, mice received Cetuximab or Cediranib, while a control group received the vehicle of the two drugs. Strikingly, we found that Cetuximab was inefficient and treated animals had tumors growing as good as in the control group. However, Cediranib treated mice showed a significant 50% (*p* = 0.003, Student *t*-test) reduction of tumor growth compared to Cetuximab and control groups. Hence, the calculated score of expression could serve to identify potential therapeutic targets in refractory tumors.

## 3. Discussion

Molecular profiling of tumors from biopsies is undoubtedly a very efficient way to get a precise diagnosis and eventually prognosis of the cancer. Large scale analyses at the genomic or transcriptomic levels have provided unprecedented knowledge on the tumor subtypes and led to the identification of biomarkers eventually predictive of a risk of cancer. Outstanding examples can be found in the detection of the BRCA1 mutation as a marker of a high risk of ovarian or breast cancer [29] or monitoring of the PSA antigen for the diagnostic of prostate cancer [30]. However, few biomarkers can be used to stratify patients with regards to their response to a treatment, for example HER2 for predicting Trastuzumab efficacy for breast cancer [31], RAS for Cetuximab, and Panitumumab for metastatic colorectal cancer [9,32], BRAF for Vemurafenib efficacy for metastatic melanoma [33]. The most common studied criteria are the genomic alterations (point mutations, deletions, insertions, and gene sequences translocations). The limit of this methodology is that a mutation is not necessarily responsible of tumor progression and/or the exact function of the genes may be poorly understood. Our study provides a novel method to analyze mRNA expression data in order to calculate a score of expression that can be used to rank target genes expression and potentially predict drug efficacy in addition to the knowledge of existing mutations being required to validate the relevance of therapeutic targets highly ranked with our procedure.

One of the main difficulties encountered when searching molecular signatures from tumor biopsies is the heterogeneity of the samples. Indeed, inter-tumor and intra-tumor heterogeneity is common and local variations of the percentage of tumor cells compared to stromal cells, immune cells or vascular cells contribute to create an extraordinary complexity. Hence, it is mandatory to use the right reference to be able to compare expression data to avoid false conclusions on the apparent deregulation of gene expression that might simply reflect the contribution of one particular cell type instead of real abnormal expression in the tumor cells. One way to take into account this complexity is to compare gene expression in the tumor sample to the one measured in the different cell types potentially composing the tumor sample. A comparison of the expression levels between the tumor and the whole organ hosting the tumor is in fact the easiest way to include all potential normal cell types that may be present in the tumor sample. This has been successfully performed to develop the OncoFinder pathway activation strength method enabling efficient prediction of patient’s response to cetuximab [34]. However, using the whole organ as a reference is only providing an average signal, masking potential variations of expression from one cell type to another. To address this point and to increase the sensitivity of the analysis, we decided to include comparisons of the level of expression in isolated major cell types composing the organ hosting the tumor. Hence, considering the importance of tissue remodeling during the process of cancer, a pre-cancerous tissue appeared interesting to catch any early significant modification of the expression profiles of target genes. Our analysis was indeed conducted by RT-qPCR when using housekeeping genes 18S and GADPH to express data (raw data) or when comparing the expression of a given gene with the one measured in the whole colon (organ hosting the tumor), in a precancerous lesion (polyp) or in two major cellular components (colon epithelial cells, smooth muscle cells) composing the organ. Importantly, we verified that only one reference sample is sufficient of each subtype. To explore this point, we have done data mining of target genes expression in 69 different parts of human normal colon biopsies has revealed that the selection of one colon sample is representative of the more general expression in a large population. However, our data showed clear differences in the determined expression levels of the target genes depending on the reference sample use to calculate the relative expression. The strongest variations were observed when comparing to the cellular components, while comparison to the organ or precancerous lesion were generally not discriminant. This strongly suggests that the use of individual cell types is more relevant than the whole normal tissue to extract gene expression variations. However, without the technical possibility to obtain all isolated cell types, it is important to keep the comparison with the whole organ, guaranteeing the largest diversity of cell types. From one sample to another, differences were not necessarily concerning the same genes. Rather, individual profiles were seen for each tumor sample. Thus, we decided to take into account the differences in the measurement of gene expression levels by determining a calculated score of expression integrating the sum of the individual expression levels in comparison to all different reference samples. To facilitate analysis and rescaling of expression data, the highest deregulated target gene expression (being the sum of the different 2^−ΔΔct^) was arbitrarily set to 1000, a value then used to express normalized scores for the other genes. In this case, expression data for a given gene resulted from a normalization process taking into account variations due to the cellular heterogeneity. Interestingly, the analysis of tumor samples containing various percentage of tumor cells and stroma showed that our method is indeed compensating/correcting the apparent expression levels as the signatures were similar while contained 20–70% tumor cells (see Figure 2). This correction of expression data was then challenged using a collection of 15 PDX models for which an objective response to Cetuximab treatment was available [28]. In this cohort, the EGFR level of expression is not predictive of a response to Cetuximab in non-normalized expression data were also not correlated with treatment efficacy in this cohort. However, the calculated expression scores were exhibiting a significant correlation with the response rate to Cetuximab treatment. Moreover, the median of the expression scores appeared as a predictor of drug efficacy, since scores above it corresponded to responding tumors, while scores below the median were not. Without objective clinical response for all of the other selected target genes in these PDX models, it is difficult to affirm that median scores of the other targets would also predict drug efficacy. However, the selection of a drug targeting the genes with the highest score of expression showed anti-tumor activity in a Cetuximab refractory model. Thus, while needing further work to identify the threshold of therapeutic efficacy for each targeted therapy, the method proved the existence of a link between the calculated scores and tumor response. To the contrary of other methods like OncoFinder [34] focused on EGFR pathway, our proof of concept experiment was conducted on a selection of nine genes arbitrary chosen because they were described for their role in colon cancer progression and being the target of well-documented drugs. The selected genes should not be considered as a definitive molecular signature with any diagnosis, prognosis, or relative drug efficacy value, but only as a model signature serving to challenge the normalization process we developed. In other words, the inclusion of additional or different genes being potentially targetable with drugs could provide very different hierarchy, thus leading to alternative therapeutic options. However, from a clinical perspective, the possibility to predict the efficacy (or non-efficacy) of anti-proliferative drugs (such as Cetuximab) or anti-angiogenic drugs (such as Cediranib) is of prime interest in the context of colon cancer for which this therapeutic option is mainly driven by the localization of the tumor [35]. Future investigations will also analyze from clinical retrospective studies the correlation between calculated scores of expression and objective response to targeted therapies. We have already conducted a clinical retrospective study on colorectal cancer, demonstrating that the VEGFA score is correlated with patient recurrence free survival and with cancer specific survival [36]. A systematic approach can now be conducted in order to show our method as a general procedure allowing the integration of tumor complexity in the determination of gene expression.

## 4. Material and Methods

### 4.1. Tumor Samples Collection

RNA from nine colon tumor stage I or IV (Z5ALYRSH Male, STAGE I, 83 years old/OQMNOR32 Male, STAGE IV, 52 years old/FC1AVRAA Female, STAGE I, 52 years old/4QDH8RIJ Female, STAGE I, 44 years old/RVBKJR34 Male, STAGE IV, 77 years old/EK21MRMMZ Male, STAGE IV, 66 years old/R5NSMRQV Female, STAGE IV, 62 years old/565HFAF2 Male, STAGE I, 77 years old/65SVOR2E Male, STAGE IV, 78 years old) were obtained from Bioserve. Collected frozen fragments from 15 established patient-derived colorectal tumors xenografted and serially passaged subcutaneously in mice were provided by Oncodesign (CR-IC-004M-P4 Male, 70 years old/CR-IC-006M-P3 Female, 45 years old/CR-IC-007M-P4 Female, 68 years old/CR-IC-009M-P3 Female, 73 years old/CR-IC-0013M-P3 Male, 68 years old/CR-IC-0021M-P4 Female, 80 years old/CR-IC-0025M-P3 Male, 60 years old/CR-IC-0028M-P3 Male, 58 years old/CR-IGR-002M-P4 Male, 69 years old/CR-IGR-0023M-P3 Female, 64 years old/CR-IGR-048M-P3 Female, 68 years old/CR-IGR-052C-P4 Male, 71 years old/CR-LRB-008M-P4 Female, 80 years old/CR-LRB-009C-P4 Female, 50 years old/CR-LRB-019C-P5 Female, 58 years old).

### 4.2. RT-qPCR

Total RNA was extracted with Tri Reagent^®^ solution (Molecular research center; RNA/DNA isolation reagent #TR118) according to manufacturer’s instructions. RNA concentration was measured with a spectrophotometer (Thermo Scientific; Nanodrop 1000) and 2 µg of total RNA/10 µL were treated with DNase-I (ROCHE) and the reverse transcription was done with High Capacity cDNA Reverse Transcription Kit (10 min at 25 °C, 2 h at 37 °C, 5 min at 85 °C) (APLLIED BIOSYSTEM #4368814). cDNA were then diluted to get a finale concentration of 1 µg/100 µL. cDNA Quantitative reverse transcriptase polymerase chain reaction (RT-qPCR) was performed using the 7500 Real time PCR System (Life technologies) with TaqMan Gene expression Master Mix (APPLIED BIOSYSTEM #4369016) with a concentration of cDNA of 10 ng in a final volume of 20 μL. Thermal cycling conditions were: 2 min at 50 °C, 10 min at 95 °C (Holding step), and 40 cycles of 15 s at 95 °C (Melting step) + 1 min at 60 °C (Annealing/Extension step). Experiments were conducted using customized microplates specially produced for this project by Applied Biosystems (APPLIED; Custom TaqMan Array Plates) to contain human specific TaqMan^®^ probes (1× final concentration is 250 nM) and primers (1× final concentration is 900 nM per primer) for: EGFR (Epidermal Growth Factor Receptor; Hs01076078_m1); FLT1 (Vascular Endothelial Growth Factor Receptor 1; Hs01052936_m1); HER2 (Human Epidermal Growth Factor Receptor-2; Hs01001580_m1; ITGB1 (Integrin Subunit Beta 1; Hs00236976_m1); KDR (Kinase Insert Domain Receptor; Hs00911700_m1); MMP2 (Matrix Metalloproteinase-2; Hs00234422_m1); MMP9 (Matrix Metalloproteinase-9; Hs00957562_m1); PDGFRA (Platelet-Derived Growth Factor Receptor, alpha; Hs00183486; VEGFA (Vascular Endothelial Growth Factor A; Hs00173626_m1). We attributed a ct value of 40 to ct that were undetermined. Relative expression level of genes was obtained by the comparison with the mean expression of two housekeeping genes, the ribosomal 18S (18S ribosomal RNA; Hs99999901_s1) and the GAPDH (Glyceraldehyde 3-phosphate dehydrogenase; Hs99999905_m1): Δct (gene) = ct (gene)–mean ct (housekeeping genes). 2^−Δct^ (gene) corresponds to RNA quantity.

### 4.3. Normalization Process

Target gene expression levels in the tumor are normalized with ΔΔCt method by several comparison to reference samples as: (i) a normal tissue, whole normal colon (ZYAGEN; HR-311) (ii) tissue cellular subtypes, human colonic smooth muscle cell (CLINISCIENCES; 2945-SC) and human colonic epithelial cells (CLINISCIENCES; 2955-SC), and a low grade or benign tumor tissue, precancerous Polyps (09H20450 Centre de Ressources Biologiques, Hôpitaux Universitaires de Strasbourg, Hôpital de Hautepierre, Strasbourg, France; gift from Dr D. Guenot). Results obtained after these different comparisons to reference samples are then added to determine an intermediate score defined as the sum of 2^−ΔΔCt^. This intermediate score is calculated for each target genes and represents the global amplitude of this target gene expression variation in the cancer sample compared to the expression in reference samples:

(1) $$intermediate\;score=\overset{n}{\underset{k=1}{\sum }}[{2^{-\Delta \Delta Ct}\left(reference\;sample\right)}_{k}]$$

where ΔΔ*Ct* (target genes) = Δ*Ct* (target genes in tumor sample) − Δ*Ct* (target genes in reference sample) and Δ*Ct* (target genes in reference sample) = Ct (target genes in reference sample) − mean of *Ct* (reference gene in reference sample)

These intermediate scores are then ranked in a decreasing order and the target gene with the highest intermediate score is arbitrary set to 1000 points. The other target genes are then normalized from this maximal value. Each expression level is therefore shown as a score of expression between [0–1000]. The scale is linear from [0–1000] for ranking and a logarithmic (Log10) is used for radar mode representation of data.

### 4.4. Heterotopic Grafting of Colorectal Cancer Patient Derived Xenograft

Animal facility is authorized by French authorities Agreement N° A21231011EA. All Experiments were performed according to the Guide for Care and Use of Laboratory Animals (E67-6-482-21) and the European Directive with approval of the regional ethical committee (Reference AL/55/62/02/13) and the Animal Care and Use Committee of Oncodesign (Oncomet, CNREEA agreement N°91). Small tumor fragments (CR-IC-0028M-P3 not responsive to Cetuximab) were subcutaneously implanted in the right flank of CB17 SCID mice. When tumor size reached 500–700 mm^3^, tumors were surgically excised and small tumor fragments were subcutaneously implanted in the right flank of 30 recipients SWISS Nude mice. The treatment started when tumors reached a mean volume of 200–300 mm^3^. The first group of the model CR-IC-028M was treated with Cetuximab 12.5 mg/kg (*n* = 10) i.p. once per week for 3 weeks, the second group was treated with Cediranib 6 mg/kg (*n* = 10) p.o. for 10 days interrupted by 2 days of wash-out after 5 days. The last one, the control group, received a mix of the vehicle of Cetuximab and Cediranib. The administration volume for the two models was 10 mL/kg (200 µL/mouse of 20 g) adjusted to the most recent individual body weight of mice. Cediranib (Selleckchem) is solubilized in methylcellulose 0.5% in PBS. Cetuximab is solubilized in NaCl 0.9% (Aguettant, Lyon, France). The tumor volume was calculated by [(a × b^2^)/2], where a is the largest tumor diameter and b the perpendicular tumor diameter measured with a caliper every 3 days.

On the day of termination (31 days after the first treatment), mice were sacrificed by cervical dislocation before tumor collection. The response to treatments is expressed by the percentage of tumor growth inhibition index (dt/dc)% = [(median Treated Tumor volume at Day 31 − median Treated Tumor volume at Day 0)/(median Control tumor volume at Day 31 − median Control tumor volume at Day 0)] × 100 where Day Y is the day of evaluation, and Day X is the day of initiation of therapy for treated [T] and control [C] tumor volumes. Of note, treatments were administrated in blind condition regarding the prediction of efficacy. The molecular signatures and the preclinical models were conducted in independent labs by different experimenters.

### 4.5. Statistics

The radar mode representations of molecular signatures were done using Excel. All statistics and other graphics were performed using GraphPad Prism 7.03. Multiple comparisons of the molecular signatures obtained with the different normalizations (Table 1) were performed with a non-parametric Friedman test on matched data. Spearman r analysis served to determine the correlation between the score of expression of EGFR and cetuximab efficacy (Figure 4). In Figure 5, ROC curve analysis served to calculate the area under the curve (AUC with 95% confidence interval) and the differences between medians of the scores were analyzed with Mann-Withney test. *t* test analysis was performed to analyze differences in tumor growth (Figure 6).

## 5. Conclusions

We have developed a simple method designed to correct the expression levels of target genes in a tumor biopsy. This method relies on rounds of normalization of the expression data with reference samples reflecting the complexity of tumor samples in term of cell contents. This proof of concept study conducted for CRC opens the possibility to extend the strategy to other solid tumors. In this case, reference samples reflecting the composition of the organ hosting the tumor will be necessary to calculate the expression scores. Moreover, this approach is not limited to the nine target genes we arbitrary selected here. Rather, it can virtually be extended to any gene thereby allowing to identify new therapeutic targets that may not be detected with classical methods. The whole approach will now need a systematic analysis challenging the relevance in clinical settings.

## Figures and Tables

**Table 1:** Summary of the target genes included in the molecular signature.

Target Gene	Biological Function		Relevance in CRC
*MMP9*	Extracellular matrix remodeling	Proliferation	[16]
*MMP2*			[16]
*ITGB1*			[17]
*ERBB2*			[18]
*EGFR*			[19]
*PDGFRA*			[20]
*VEGFA*		Angiogenesis	[21]
*Flt1*			[22]
*KDR*			[23]

**Table 2:** Summary of the statistical analysis conducted on the molecular signatures after single normalization. Each individual signature obtained after single normalization was compared as matched data. The two by two comparisons showed non-significant variations (ns), or significant variations (* *p* < 0.05; ** *p* < 0.01; *** *p* < 0.001; Mann-Whitney test) demonstrating the importance of the normalization in getting relevant and consistent expression data. NORM = whole normal colon; SMC = smooth muscle cells; EPIC = colon epithelial cells; POL = precancerous polyp.

Sample.	Z5ALYRSH			FC1AVRAA			OQMNOR32		
Reference	SMC	EPIC	POL	SMC	EPIC	POL	SMC	EPIC	POL
NORM	**	*	ns	***	*	ns	****	ns	ns
SMC		ns	*		*	**		*	**
EPIC			ns			ns			ns
Sample	4QDH8RIJ			RVBKJR34			EK21MRMZ		
Reference	SMC	EPIC	POL	SMC	EPIC	POL	SMC	EPIC	POL
NORM	***	ns	ns	***	ns	ns	**	ns	ns
SMC		*	***		ns	*		*	****
EPIC			ns			ns			*
Sample	R5NSMRQV			565HFAF2			65SVR2E		
Reference	SMC	EPIC	POL	SMC	EPIC	POL	SMC	EPIC	POL
NORM	***	ns	ns	***	ns	ns	****	**	ns
SMC		**	***		ns	***		ns	***
EPIC			ns			ns			*

## Supplementary Materials

The following is available online at https://www.mdpi.com/2072-6694/12/1/149/s1, Table S1: Molecular characteristics of the PDX cohort.
