# Supplementary material for: Prediction of Drug Efficacy in Colon Cancer Preclinical Models Using a Novel Ranking Method of Gene Expression
Source: Cancers (Basel). 2020 Jan 8;12(1):149. doi: 10.3390/cancers12010149 (PMC7016638; doi:10.3390/cancers12010149)
Supplement: Supplementary file 1 [file cancers-12-00149-s001.pdf]

Supplementary Materials

Prediction of Drug Efficacy in Colon Cancer Preclinical Models Using a Novel Ranking Method of Gene Expression

Justine Fritz, Olivier Lefebvre, Aurore Fernandez and Jordane Schmidt and Dominique Bagnard

Table S1. Molecular characteristics of the PDX cohort.

PDX	APC	CTNNB1	KRAS	BRAF	PIK3CA	AKT1	EGFR	FBXW7	TP53	MSI	Patient's Treatment
CR-IC-004M-P4	Mut	wt	Mut	wt	wt	wt	wt	wt	Mut	MSS	FOLFIRI 6 cycles => surgery => FOLFIRI 6 cycles
CR-IC-006M-P3	wt	wt	wt	wt	wt	wt	wt	wt	Mut	MSI-L	surgery and after surgery: FOLFOX + AVASTIN 9 cycles
CR-IC-007M-P4	Mut	wt	Mut	wt	wt	wt	wt	wt	Mut	MSS	before surgery: Campto-Erbitux => surgery => after surgery: Monotherapy FUFOL (6 cycles); Chemotherapy and biotherapy) AVASTIN (6 cycles)
CR-IC-009M-P3	Mut	wt	wt	wt	wt	wt	wt	wt	wt	MSS	surgery and after surgery: Chemotherapy and bitherapy – FOLFOX6 ,2 injections in 1 cycle
CR-IC-0013M-P3	Mut	wt	Mut	Mut	wt	wt	wt	wt	wt	MSI-L	surgery and after surgery: Chemotherapy (Bitherapy): FOLFOX (6 cycles)
CR-IC-0021M-P4	wt	wt	wt	wt	wt	wt	wt	wt	Mut	MSS	surgery and after surgery: XELOX
CR-IC-0025M-P3	Mut	wt	wt	wt	wt	wt	wt	wt	Mut	MSS	surgery and after surgery: 5 cycles of FOLFOX
CR-IC-0028M-P3	wt	wt	wt	wt	wt	wt	wt	wt	Mut	MSS	surgery and after surgery: FOLFOX 6 cycles
CR-IGR-002C-P4	wt	wt	wt	wt	wt	wt	wt	wt	Mut	MSI-L	surgery and after surgery: 11 cycles of FOLFOX with reduced concentration
CR-IGR-0023M-P3	Mut	wt	Mut	wt	Mut	wt	wt	wt	Mut	MSS	before surgery: FOLFIRI + Avastin® (3 cycles) then FOLFIRI (1 cycle) => surgery
CR-IGR-048M-P3	wt	wt	wt	wt	wt	wt	wt	wt	Mut	MSS	surgery and after surgery: 3 cycles of intra-arterial l-OHP) + systemic LV5FU2 + Cetuximab
CR-IGR-052M-P4	wt	wt	wt	wt	wt	wt	wt	wt	Mut	MSI-L	before surgery: FOLFOX (11 cycles) followed by campto-erbitux (5 cycles) => surgery + 2 cycles of campto + erbitux followed by FOLFOX + erbitux
CR-LRB-008M-P4	wt	Mut	Mut	wt	Mut	wt	wt	wt	wt	MSS	before surgery: Erbitux + Avastin – more than 10 cycles => surgery => after surgery: Avastin
CR-LRB-009C-P4	wt	Mut	Mut	wt	Mut	wt	wt	wt	wt	MSS	before surgery: FOLFOX + erbitux followed by FOLFIRI + erbitux => surgery => after surgery: Avastin alone
CR-LRB-019C-P5	Mut	wt	wt	wt	wt	wt	wt	wt	Mut	MSS	before surgery: LV5FU followed by Xeloda followed by FOLFOX => surgery with Intra-peritoneal hyperthermic oxaliplatin / irinotecan / systemic FUFOL => after surgery: 4 cycles of FOLFOX

© 2020 by the authors. Licensee MDPI, Basel, Switzerland. This article is an open access article distributed under the terms and conditions of the Creative Commons Attribution (CC BY) license (http://creativecommons.org/licenses/by/4.0/).
